# Reconstructing ice-margin retreat using delta morphostratigraphy

**DOI:** 10.1038/s41598-017-16763-x

**Published:** 2017-12-05

**Authors:** Pierre Dietrich, Jean-François Ghienne, Alexandre Normandeau, Patrick Lajeunesse

**Affiliations:** 1 Institut de physique du Globe de Strasbourg, UMR 7516 CNRS/Université de Strasbourg, 1 rue Blessig, 67084 Strasbourg, France; 2 Department of Geology, P.O. Box 524 Auckland Park 2006, Auckland Park Kingsway Campus, University of Johannesburg, Johannesburg, South Africa; 3 Geological Survey of Canada–Atlantic, 1 Challenger Drive, Dartmouth, Nova Scotia, B3B 1A6 Canada; 4 Centre d’études nordiques & Département de géographie, Université Laval, 2405 Rue de la Terrasse, Québec, Québec, G1V 0A6 Canada

## Abstract

The paleogeographic reconstruction of the successive inland positions of a retreating ice sheet is generally constrained by mapping moraines. However, deltaic complexes constructed by sediment-charged meltwater can also provide a record of the retreating ice-margin positions. Here, we examine a serie of ice-contact, ice-distal glaciofluvial and paraglacial depositional systems that developed along the Québec North Shore (eastern Canada) in the context of falling relative sea level during the northward retreat of the Laurentide Ice Sheet (LIS). Ice-contact depositional systems formed when the LIS was stillstanding along the Québec North Shore. Subsequent inland retreat of the ice margin generated glacial meltwaters feeding sediment to glaciofluvial deltas, leading to their rapid progradation. The retreat of the ice margin from drainage basins was marked by the onset of paraglacial processes such as the shutdown of delta progradation, severe fluvial entrenchment, and deposition of shallow-marine strata. Four end-member scenarios describe the spatial and stratigraphic distribution of these three depositional systems (ice-contact deposits, ice-distal glaciofluvial deltas, and paraglacial suites). They reflect both the inherited drainage basin physiography and the retreat pattern of the ice margin. Applied to twenty deltaic complexes, these end-members allowed us to refine the model of LIS-margin retreat over southeastern Québec.

## Introduction

The pattern of final inland glacial retreat of ice masses is generally less constrained than those tied to earlier, marine-based deglacial stages that offer better constrained temporal framework^1–3^. Final deglaciation relates, however, to major continental-scale re-organization of ice flows and drainage basins as well as to global climate change. In eastern North America, fragmentation of the Late Wisconsinan Laurentide Ice Sheet (LIS) and drainage of Lake Agassiz are suspected to have triggered the 8.2 ka cooling event^4^. The subsequent disintegration of the residual Québec-Labrador ice sector probably had a major impact on climate and ocean circulation^5,6^. Defining the final LIS retreat pattern and chronology is thus a crucial issue when deciphering Early Holocene climate forcings.

Morainic complexes, eskers and glacial lineations deposited during the retreat of the Québec-Labrador ice sector are well-expressed landforms^1,3,7–9^ but with poorly constrained chronologies awaiting systematic direct dating on the basis of cosmogenic exposure ages^2^ and luminescence^10^. In contrast, marine and coastal depositional systems tied to earlier deglaciation stages are characterized by detailed temporal framework due to the abundance of datable material^11–13^. In particular, delta morphostratigraphies that are in part controlled by change in meltwater and sediment supply, itself intimately tied to the history of ice-margin retreat throughout drainage basins^14–17^, constitute integrative systems which the unravelling delivers crucial information about their formative processes and penecontemporaneous deglacial evolution. Indeed, such sedimentary archives have a great potential to provide additional and independent age constraints for ice-sheet retreat history in delta river drainage basins.

**Figure 1:**
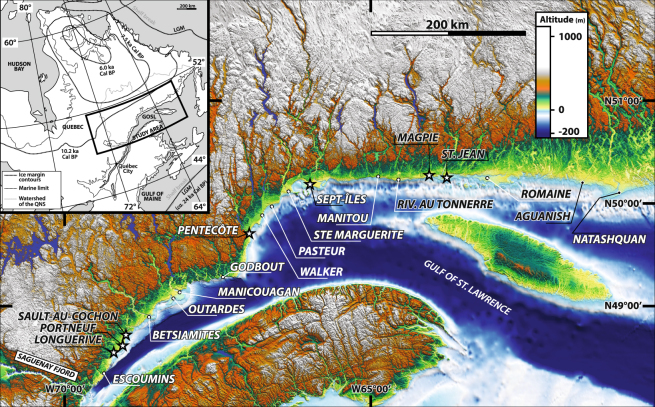
Location of the 20 deltaic complexes of the QNS in eastern Canada. Stars represent deltaic complexes with morphostratigraphies that have been more specifically investigated (Figs 2 and 3). Insert: reconstruction of ice-front positions of the Québec-Labrador Ice sector from the LGM to 6 ka^3,13^. GOSL: Gulf of St. Lawrence; QNS: Québec North Shore; LGM: Last Glacial Maximum. The background topography is from SRTM3 (ref.^14^) available at https://www2.jpl.nasa.gov/srtm/. The map was generated using GlobalMapper^®^ GIS software.

Here we integrate geomorphological and sedimentological results from twenty deltaic complexes constructed since deglaciation along the Québec North Shore (QNS, NW Estuary and Gulf of St. Lawrence). Interpreting deltaic complexes along ~1500 km of coastline allows us to map the positions of Late Pleistocene–Early Holocene inland ice margins of the retreating Québec-Labrador ice sector of the LIS during the progressive deglaciation of the adjacent drainage basins of these deltas, together representing an area of more than 250,000 km^2^ (Fig. 1).

## Deglacial history

**Figure 2:**
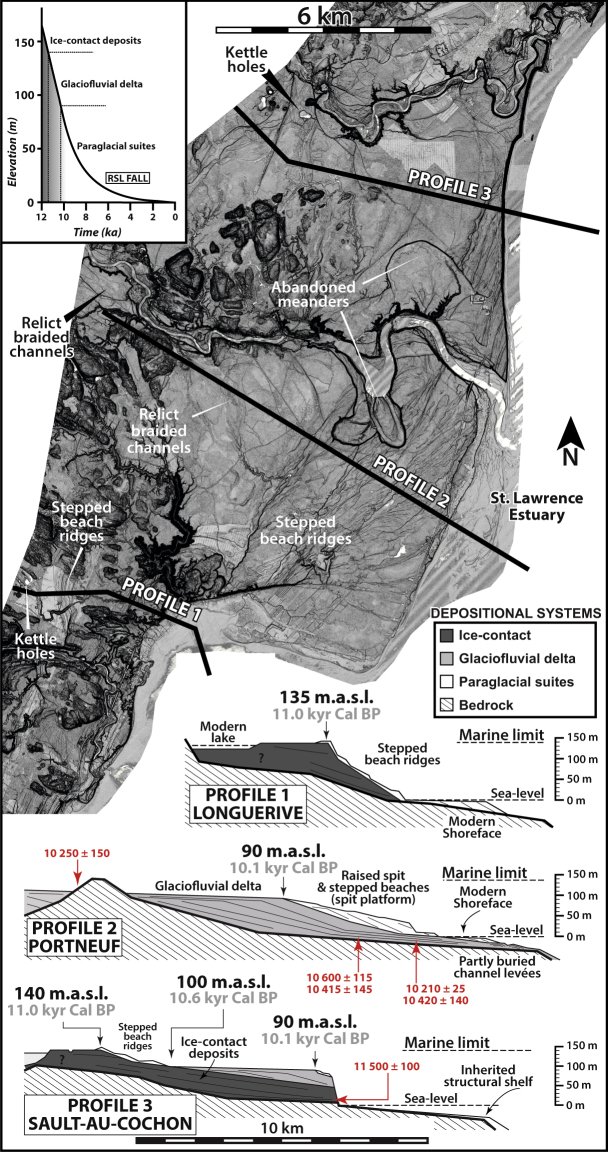
LiDAR imagery of morphostratigraphic architectures and imbrications of the three depositional systems (ice-contact, ice-distal glaciofluvial delta and paraglacial suites) constitutive of the Portneuf, Sault-au-Cochon and Longuerive deltaic complexes^20^. The insert represents the RSL fall of the study area. The LIDAR map was generated using GlobalMapper^®^ GIS software.

Following the Last Glacial Maximum (LGM) *ca*. 24 ka (ky cal BP) ago, the LIS began to recede by collapse and iceberg calving^13^ (Fig. 1). By *ca*. 12 ka, the LIS margin stabilized over the QNS^13,18^. Then, the retreat was mainly achieved by ablation of land-based glaciers up to the final disappearance of the Québec-Labrador ice sector at ~6 ka^1,9,19^. Meltwaters and glaciogenic material fed deltaic complexes positioned at the mouths of steep-flanked structural valleys and distributed along formerly glacio-isostatically flexured lowlands of the QNS (Fig. 1). These valleys hence formed fjords immediately after the ice margin retreated and postglacial sea invasion. Marine limit, i.e., the highest elevation reached by marine invasion on the glacio-isostatically depressed land, is recorded ~150 m (asl) in the area (Fig. 1) and large strips of now-emerged land expose sets of down-stepping landforms and deposits that have been recording the contemporaneous glacio-isostatic rebound and deltaic developments. Related thick deltaic successions emplaced in a context of RSL fall (Fig. 2) accumulated as long as the drainage basin remained connected to the retreating LIS margin^3,17,20^.

## Depositional systems

Deltaic complexes of the QNS typically show three superimposed or laterally juxtaposed depositional systems: 1) ice-contact deposits, formed by ice-contact subaqueous fan that sometimes evolved as ice-contact delta, 2) ice-distal glaciofluvial delta, and 3) paraglacial suites^20^. Although the deltaic complexes of the QNS are characterized by a common deglacial framework and RSL fall history —based on radiocarbon dating—, they may vary in terms of depositional architectures. Figure 2 outlines three archetypal morphostratigraphies of the QNS deltaic complexes^20^. As related depositional systems have specific and unique morphological and surficial expressions, they can confidently be identified on satellite and aerial imagery and/or on digital elevation models.

Ice-contact deposits represent volumetrically massive sediment wedges forming the core of some of the deltaic complexes. They were constructed immediately after local deglaciation during major ice-marginal stillstands at the outlet of structural valleys that have most likely been favored by bedrock thresholds^21^. Note that backstepping grounding zone wedges may in some case have been emplaced at earlier —and deeper— stillstand positions^18,22^. Ice-contact subaqueous fan and ice-contact delta architecture includes boulder-bearing pebbly topsets^20^ deposited from either subaqueous or subaerial meltwater outflows^23^, and foreset beds alternating siltstones and thick graded sand beds deposited by low- and high-density turbidity currents, respectively^24^, and commonly supercritical^25^. The topmost surface of ice-contact deposits is flat to very gently-sloped (<1°) and stands at or slightly below the marine limit (Fig. 2). The subsequent retreat of the ice margin forced the abandonment of the ice-contact depositional system and led to its reworking by shore-related processes through the ongoing RSL fall.

Ice-distal glaciofluvial deltas refer here to deltas fed by a fluvial network issued from the retreating ice margin and where the hydraulic regime was adjusted to sediment-charged meltwater flows^26,27^. Initially confined within the structural valleys invaded by the sea (fjord-head deltas), glaciofluvial deltas rapidly expanded on the St. Lawrence coastal shelf owing to high rates of delta progradation^17,20^. Even though the ice margin might have been located tens of kilometers further inland, sediments were transferred to the coast throughout the structural valleys *via* the riverine network and permitted a perennial delta accretion. The stratigraphic architecture of glaciofluvial deltas typically shows flat- to gently-sloped topsets deposited by braided streams, upper foresets consisting of stacked graded sand beds deposited by high-density turbidity current, and gently-sloped lower foresets formed by low-density turbidity currents. In spite of high rates of RSL fall forced by the glacio-isostatic rebound^28^, no coeval fluvial entrenchment was identified at least in the early part of this stage. The absence of incision in pre-existing sediments is due to the large amount of glacially-derived sediment supply that maintained a fluvial equilibrium profile steeper than the shoreline trajectory^20,29–31^. The extensive (several tens of km²) top surfaces of the glaciofluvial deltas, which locally preserve relict braided channels and isolated beach ridges, stand well below marine limit (100–20 m asl, Fig. 2).

Paraglacial suites include stepped coastal deposits and meander belts, which the onset arose when the ice margin melted out of the river drainage basins. Paraglacial suites hence correspond to the product of the reworking of the then inactive glaciofluvial delta due to the shutdown of glaciogenic sediments previously delivered to the coast (i.e., middle to late paraglacial *sensu* Hein *et al*.^16^). Coastal deposits consist of stepped beach and shoreface wedges deposited along inherited foreset slopes of the ice-contact deposits and/or glaciofluvial delta^20^. Occasionally, coastal deposits forming thicker (>15 m) sand wedges correspond to coastal spit platforms characterized by composite offlapping geometries (Fig. 2). Coastal deposits are genetically associated with incised meander belts, which the formation resulted from the entrenchment and repeated channel avulsions of the fluvial system within the abandoned glaciofluvial delta throughout the ongoing RSL fall. Surficial expression of coastal deposits and meander belts are well-recognized as they form stepped, linear or curvilinear sand ridges, paleocliffs, spits and cusps, and stepped and abandoned meandering fluvial terraces, respectively (Fig. 2).

Similar sedimentological and stratigraphic evolutions built up by ice-margin retreat and associated RSL fall forced by the glacio-isostatic rebound have been observed elsewhere throughout Quaternary successions^32^, in particular in Greenland^33^, Scandinavia^34–36^ as well as in the Hudson Bay^37,38^.

## Evolution of deltaic complexes

**Figure 3:**
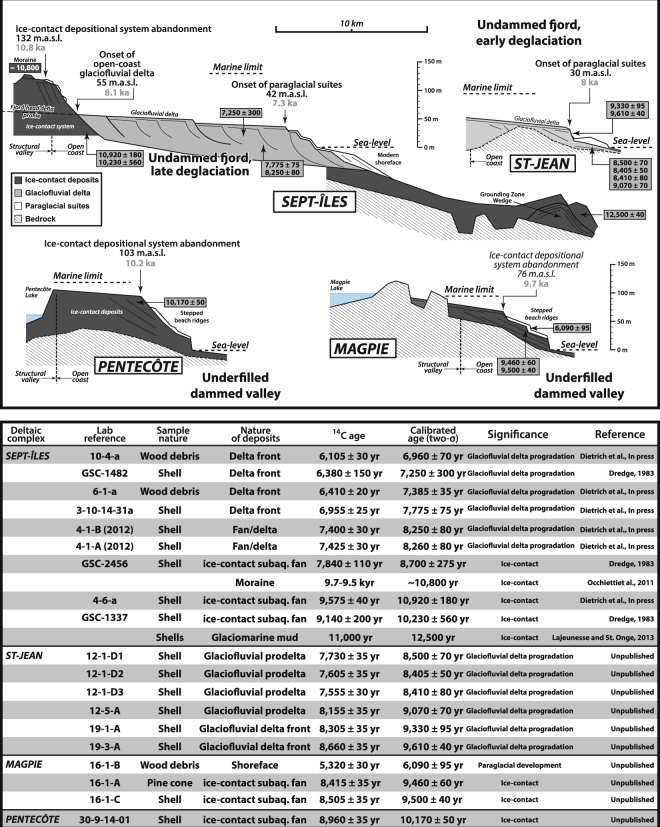
Morphostratigraphies of four other deltaic complexes that have been investigated (locations, Fig. 1), for which the temporal framework is derived from direct radiometric dating of the landforms and/or deposits. Further details for the Sept-Îles complex are available in Dietrich *et al*.^42^.

**Figure 4:**
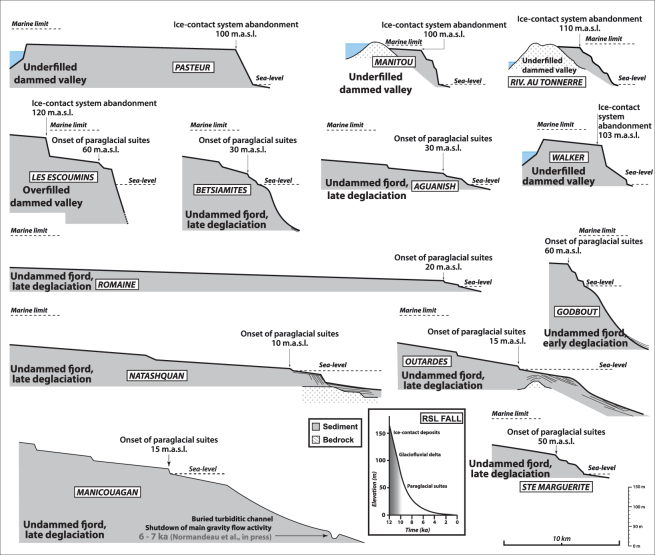
The thirteen other deltaic complexes along the Québec North Shore for which no or limited sedimentological and stratigraphic datasets are available. The top surfaces of the ice-contact depositional systems are recognized owing to their proximity with the marine limit; the top surfaces of glaciofluvial deltas are delineated based on the gently-sloped extensive delta plain distinctively lying well-below the marine limit; and, paraglacial suites are characterized by steep-sloped deposits consisting of stepped terraces. Ages of the geomorphological features were extrapolated from their elevation providing local relative sea level curves that can be inferred from isobase maps^13^. Altitudes of the marine limit were either derived from local relative sea level curve or geological/geomorphological maps when available^7,44,47–49^.

**Figure 5:**
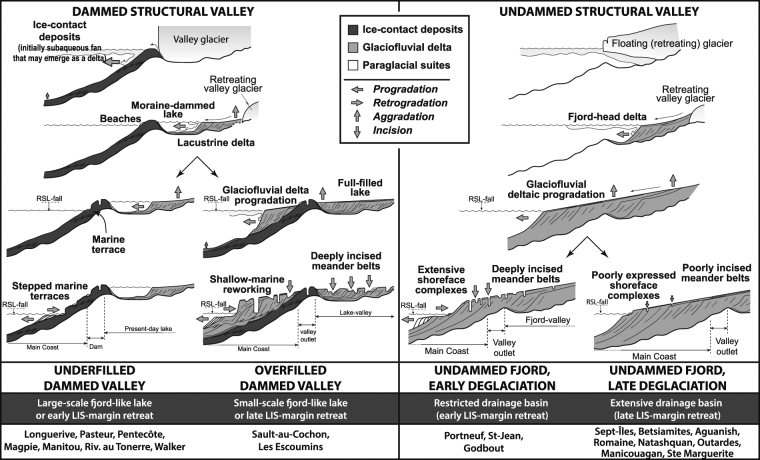
The four end-members scenarios of morphostratigraphic development of deltaic complexes along the Québec North Shore depending on ice margin retreat history and inherited topographies (see text for details).

Stratigraphic relationships between the three above-described morphosedimentary systems arise in a generic architectural framework, which have been investigated from a selection of seven deltaic complexes (Figs 1, 2 and 3). This stratigraphic framework has been expanded and adapted to the entire QNS based on aerial and satellite photographs, LiDAR and digital elevation model datasets, as each of the three type of depositional system has a specific morphological expression allowing to confidently identify them on aerial imagery (Fig. 2). Thus, thirteen additional deltaic complexes are accounted for (Fig. 4), for which the ages of landforms and deposits have been essentially extrapolated from regional RSL curves^13^ (Fig. 2 and Table 1). The diversity of observed stratigraphic architectures is categorised into four end-member scenarios representative of combined interactions between patterns of ice-margin retreat and inherited topographies (e.g., valley depths, extent of drainage basins, Fig. 5).

**Table 1:** Altitudes and related ages of the landforms observed throughout the 20 deltaic complexes investigated along the Québec North Shore and used to reconstruct the pattern of the Laurentide Ice Sheet margin retreat presented in Fig. 4.

Deltaic complex	Altitude	Ages (kyr cal BP)	Depositional system	Latitude	Longitude
Les Escoumins	120	12.5	Ice-contact system	N48°21′	W69°23′
	60	9.5	Glaciofluvial delta		
Longuerive	135	11	Ice-contact system	N48°34′	W69°17′
Portneuf	90	10.1	Glaciofluvial delta	N48°38′	W69°06′
Sault-au-Cochon	140	11	Ice-contact system	N48°42′	W69°05′
	90	10.1	Glaciofluvial delta		
Betsiamites	30	8	Glaciofluvial delta	N48°54′	W68°39′
Outardes	15	7.5	Glaciofluvial delta	N49°08′	W68°17′
Manicouagan	15	7.5	Glaciofluvial delta	N49°08′	W68°17′
Godbout	60	9.5	Glaciofluvial delta	N49°19′	W67°36′
Pentecôte	103	10.2	Ice-contact system	N49°46′	W67°11′
Walker	103	11.1	Ice-contact system	N49°56′	W67°02′
Pasteur	100	10.9	Ice-contact system	N50°02′	W66°57′
Ste. Marguerite	50	8.2	Glaciofluvial delta	N50°10′	W66°38′
Sept-Îles	130	10.8	Ice-contact system	N50°15′	W66°09′
	42	7.3	Glaciofluvial delta		
Manitou	110	12.5	Ice-contact system	N50°18′	W65°15′
Riv. Au Tonnerre	110	12.5	Ice-contact system	N50°17′	W64°55′
Magpie	76	9.7	Ice-contact system	N50°20′	W64°25′
St. Jean	50	8	Glaciofluvial delta	N50°18′	W64°11′
Romaine	20	7.5	Glaciofluvial delta	N50°17′	W64°26′
Aguanish	30	8.5	Glaciofluvial delta	N50°13′	W62°07′
Natashquan	10	7.5	Glaciofluvial delta	N50°09′	W61°46′

Ages of the “*Ice-contact system*” relate to their abandonment succeeding to a glacial stillstand and ages of “*Glaciofluvial delta*” refer to the melting of the Laurentide Ice Sheet margin out from the drainage basin of the related delta.

### Underfilled dammed valley

In this scenario, an extensive ice-contact depositional system initially develops at the outlet of the valley on a bedrock threshold during a stillstand of the ice margin. Sediment aggradation combined with subsequent RSL fall may ultimately result in development of an initial ice-contact subaqueous fan into an ice-contact delta depending on sediment availability, duration of glacial stillstand and RSL fall rates^34,39^. After further ice-margin retreat from its temporary stable position at the valley mouth, the coarse-grained fan —or any bedrock sill on which the fan was anchored— dams a fjord lake at the head of which a lacustrine ice-distal glaciofluvial delta develops^21,39^. The foresets of the then-abandoned ice-contact fan or delta are actively reworked by shallow-marine processes through the ongoing RSL fall. The lake remains underfilled of sediment^40^ either because it is too voluminous to be filled or/and owing to an early LIS-margin retreat from the drainage basin.

In the *Underfilled dammed valley* scenario, beach ridges and marine terraces are stepped from the top of the ice-contact depositional system down to modern sea level. As the lake acts as a perennial sediment trap, any delta development over the coastal shelf has been inhibited up to today, as lakes still exist nowadays. The Longuerive, Pentecôte and Magpie deltaic complexes, and which lakes are dammed by boulder-rich ice-contact deposits potentially lying on bedrock threshold, relate to this first end-member (Figs 1, 2 and 3). Lakes dammed by both Quaternary deposits and bedrock sills are also included in this scenario (e.g., Manitou or Lake St. Jean^41^).

### Overfilled dammed valley

The *Overfilled dammed valley* scenario is initially similar to the *Underfilled dammed valley* scenario, namely the development of an initial ice-contact depositional systems at the valley outlet and then of a fjord lake. However, a significant and/or protracted glaciofluvial sediment supply relative to the lake volume subsequently results in the complete infill of the lake. Sediment supply allows the fluvial system to be re-established up to the open coast through the entire valley. The afterward retreat of the LIS margin from the drainage basin forces the fluvial entrenchment and the cannibalization of ice-contact and glaciofluvial sediments by coastal processes.

In the *Overfilled dammed valley* scenario, fjord-mouth deltaic complexes show an initial outwash fan where the lower reach of its foreset slope is buried beneath glaciofluvial deltaic sediments. The elevation difference between the top surfaces of the outwash fan and the glaciofluvial delta reflects the duration of the lake infill according to the local RSL curve. The archetypal delta of this scenario is the Sault-au-Cochon deltaic complex^20^ (Fig. 2).

### Undammed fjord, early deglaciation

In this scenario, no ice stillstand occurs at the valley mouth (absence of sedimentary deposits and/or bedrock sill?). As a consequence, the ice margin retreats inland, turning the valley into a fjord during marine invasion^36^. The glaciofluvial delta then rapidly progrades into the fjord, allowing the filling of the entire valley down to its outlet. Rapid deglaciation of the river drainage basin, owing to local high rates of ice-margin retreat and/or a restricted drainage basin area, is responsible for an early transition to paraglacial conditions that consequently occurs at a RSL well above (>60 m) the modern shoreline.

In the *Undammed fjord*, *early deglaciation* scenario, as no ice-contact system has been deposited at the fjord mouth, the deltaic complex is twofold, only comprised of a glaciofluvial delta overlain and reshaped by long-lasting paraglacial processes that resulted in well-developed paraglacial suites that include severely entrenched meander belts and stepped marine terraces. The elevation difference between marine limit and the top surface of the glaciofluvial delta relates to the duration of the in-valley deltaic progradation before expanding onto the open coast domain. Such systems are typified by the Portneuf and St. Jean River deltas (Figs 2 and 3).

### Undammed fjord, late deglaciation

In this fourth and last scenario, the transition to paraglacial condition is delayed compared with the previous scenario owing to a large drainage basin and/or low rates of ice margin retreat throughout the drainage basin. Restricted paraglacial suites have hence been emplaced. Although the modern meandering river is entrenched, abandoned meander belts are not identified as the fluvial channel remained confined within the same pathway. The Sept-Îles deltaic complex, one of the largest depositional systems of the QNS, is emblematic of the *undammed fjord*, *late deglaciation* scenario. It is associated with a drainage basin extending far to the north^17,42^.

## Deltaic complexes as a proxy for reconstructing ice-margin retreat history

**Figure 6:**
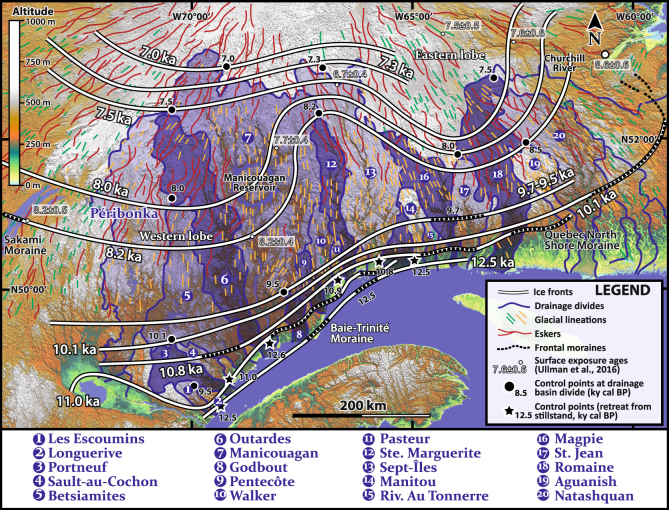
Reconstruction of the Laurentide Ice Sheet margin retreat through river drainage basins inferred from deltaic morphostratigraphies reconstructed from control points deduced from either ice-contact depositional system abandonment (stars) or onset of paraglacial suites development (melting of the ice margin out of the drainage basin, dots). See Nutz *et al*.^41^ for deglacial pattern around Lake St. Jean. Our 8.2 ka ice margin correlates with the Sakami moraine^3^ and the 8.6 ka ice margin of Carlson *et al*.^19^. Surface exposure ages derive from Ullman *et al*.^6^. Esker network and glacial lineations originate from Klassen *et al*.^7^, Fulton *et al*.^44^ and Clark *et al*.^1^. See Fig. 1 for details on the background topographic map (SRTM3^14^).

The morphostratigraphies of deltaic complexes are thus intrinsically linked to the retreat pattern of the ice margin since the latter has controlled meltwater and sediment discharges and, eventually, specific depositional scenarios (Fig. 5). Hence, combining the age constraints derived from deltaic morphostratigraphies with published records of morainic complexes (Québec North Shore, Baie Trinité, Sakami…)^1,3,7,8^, submerged grounding zone wedges^18,22^ and surface exposure ages^2,6^, we here refine the reconstruction of LIS retreat from the coastal QNS up to more than 400 km inland (Fig. 6).

When present, ice-contact deposits lying on the QNS provide information on the time interval corresponding to LIS-margin stillstand at the valley outlet, either from altitude of the top surface (date inferred from RSL curve) or radiocarbon dates (black stars on Fig. 6). Age constraints and the occurrence of glacial stillstands are corroborated by occurrences of frontal moraines (dotted lines on Fig. 6). Furthermore, the age of the deglaciation of a given drainage basin is inferred from the morphostratigraphic deltaic record lying at its outlet by the age of the transition from a glaciofluvial delta progradation to a paraglacial setting (black dots on Fig. 6). Twenty control points have hence been obtained in the QNS hinterland as this scheme has been applied for each basin draining into the St. Lawrence. Control points with the same ages have been further linked together to draw reconstructed positions of the retreating ice margin (Fig. 6). For example, an age of 8.0 ky obtained at the head of the St. Jean drainage basin has been correlated with the 8.0 ky found at the head of the Betsiamites basin, keeping in mind that the intermediate Ste. Marguerite and Manicouagan basins partake different (younger) ages. The method permits a reconstruction of ice-margin configurations (Fig. 6). We are aware that the past extent of the drainage basins may have been different from the present-day configuration due to a differential glacio-isostatic flexure^43^ (*ca*. 1 m.km^−1^). We argue, however, that this error can be considered as negligible as the drainage divides lie in relatively high-relief areas.

Our reconstruction then shows linear margins from 12.5 to 9.5 ka which agrees with the overall deglaciation framework of Occhietti *et al*.^3^, although two 100s km-wide lobate ice margins are delineated for the period 8.2–7.0 ka (Fig. 6). Lobate geometries have been deciphered from the morphostratigraphic record of the Ste. Marguerite River, indicating an early deglaciation of its drainage basin relative to the two adjacent ones. Indeed, the Romaine River drainage basin, to the east, and the Péribonka^41^, Betsiamites and Outardes drainage basins, to the west, were fed by meltwaters until 7.5–7.0 ka. In addition to headland deglaciation points, the proposed ice-margin reconstruction at 8.2–7.0 ka is coherent with esker distribution maps^7,9,44^ (Fig. 6), provided that eskers develop near and perpendicular to the retreating ice margin^45^. The inferred lobate ice margin is a reminiscence of mapped ice-stream lobes^1^, as both eastern and western lobes fit fairly well with the 8.0 ka-aged part of the Flow Set 23 reconstruction of Clark and co-authors^1^. In contrast, glacial lineations^1,7,44^ (Fig. 6) are likely to reflect older ice flow configurations prevailing either during the less-constrained 9.5–8.2 ka interval or possibly even before 12.5 ka as these landforms are essentially tangential to the inferred ice-margin positions during the 8.2–7.0 ka interval. Further, surface exposure ages^6^ corroborate our model of ice-margin retreat, either for the western lobe over the Manicouagan drainage basin. Our 8.2 front matches their 8.2 ± 0.4 age obtained south of the Manicouagan reservoir while their 7.7 ± 0.4 agrees with our 8.0 ice front north of the reservoir. Similarly, their ages obtained in the Churchill River in the northeastern part of the study area fit with our reconstructed ice margins (Fig. 6).

Our deglaciation model indicates low retreat rates of the older, linear ice margin from 12.5 to 9.7 ka. The older dates correspond to the Younger Dryas (YD) Cold Event dated from 12.8 to 11.7 ka, and bracketed further south by the Mars-Batiscan (12.2 to 11.5 ka) and St. Narcisse (12.8 to 11.5 ka) moraines, the latter being likely equivalent to the Québec North Shore moraine^3^. If related to the YD, the slow retreat of this old linear ice margin should reflect at least in part the signature of a global climatic cooling. The shift from linear ice margin to lobate features (ice-stream lobes) between 9.7 and 8.2 ka likely reflects the transition from frozen to warm basal thermal conditions of the Québec-Labrador ice sector which could be associated with a phase of development of ice streams^1^. This cold to warm based ice transition occurring slightly after 10 ka according to these authors. This hypothetical cold- to warm-based transition is in agreement with Tarasov and Peltier^46^ who suggest an abrupt increase of the proportion of warm-based ice at 8 ka. Between 9.7 and 8.2 ka, a rapid (130 m.yr^−1^) retreat rate characterizes the Ste. Marguerite inter-lobe area that subsequently decreased down to 80 m.yr^−1^ between 8.2 and 7.0 ka. Comparatively, retreat rates of 80 m.yr^−1^ and 160 m.yr^−1^ took place throughout this entire period for the eastern and western lobes, respectively. Compared to the post-8 ka enhanced retreat (900 m.yr^−1^) corresponding to periods of enhanced melting^2,19^, our reconstruction shows more steady overall rates of retreat during the LIS deglaciation.

The correspondence between our morphostratigraphy-based reconstruction and other published reconstructions is a validation of the here-proposed method, which thus appear robust as far as a great number of deltaic complexes have been integrated into our model. Our reconstruction complements and strengthens previous ice-margin retreat models based on glacial landforms and surface exposure ages. However, our aim here is to provide a method for constraining retreat patterns solely based on delta morphostratigraphies which, beyond the particular case of the LIS retreat in south-eastern Québec, should be applicable to other studies of formerly glaciated regions. Particularly, the deltaic systems of eastern Hudson Bay were supplied in sediments by an extensive drainage basin formerly occupied by the northern Québec-Labrador residual ice sector almost up to its final disintegration (coastal deglaciation around 8 ka, glacial retreat after 7 ka^37^). This model can also be further expanded to deltaic complexes lying in the eustatically-dominated zone (Newfoundland, Gulf of Maine) for which the morphostratigraphic record of ‘lowstand deltas’ is preserved at depth owing to the postglacial eustatic transgression^16^. Our approach of reconstructing past ice-margin configurations from the delta morphostratigraphic record can provide valuable inputs to data-calibrated models to infer the general retreat pattern of the LIS^43^.
